# Drug therapy-related problem management in Nigeria community pharmacy – process evaluation with simulated patient

**DOI:** 10.1186/s12913-022-07535-z

**Published:** 2022-02-16

**Authors:** Showande Johnson Segun, Lawal Sodiq Damilola

**Affiliations:** Department of Clinical Pharmacy and Pharmacy Administration, Faculty of Pharmacy, University of Ibadan, Ibadan, Nigeria

**Keywords:** Drug therapy-related problems, Community pharmacy, Pharmacist, Simulated patient, Nigeria

## Abstract

**Background:**

Unresolved drug therapy-related problems (DTRPs) have economic and clinical consequences and are common causes of patients’ morbidity and mortality. This study evaluated the ability of community pharmacists to identify and resolve DTRPs and assessed the perceived barriers to DTRP identification and resolution.

**Methods:**

A cross-sectional study which employed the use of three simulated patients (SPs) visit to 36 selected community pharmacies in 11 local government areas in Ibadan, Nigeria. The SPs played the role of a patient with prescription for multiple ailments (23-year-old male), type 2 diabetes and hypertensive patient with medication packs (45-year-old male) and hypertensive patient with gastric ulcer with a prescription (37-year-old female). They re-enacted three rehearsed vignettes when they spoke with the pharmacists. A five-member panel of experts predetermined the DTRPs present in the vignettes (*n* = 11), actions to take to investigate the DTRPs (*n* = 9) and recommendations to resolve the DTRPs (*n* = 9). Pharmacists’ perceived barriers to the identification and resolution of DTRPs were assessed with a self-administered questionnaire. The percentage ability to detect and resolve DTRPs was determined and classified as poor ability (≤30%), fair ability (> 30 - ≤50%), moderate ability (> 50 - ≤70%) and high ability (> 70%).

**Results:**

One hundred and eight visits were made by the three SPs to the pharmacies. In total, 4.42/11 (40.2%) DTRPs were identified, 3.50/9 (38.9%) actions were taken, and 3.94/9 (43.8%) recommendations were made to resolve the identified DTRPs. The percentage ability of the community pharmacists to detect and resolve DTRPs varied slightly from one vignette to another (vignette 1–49.3%, vignette 2–39.1%, vignette 3–38.8%). But overall, it was fair (40.9%). Pharmacists’ perceived barriers to DTRP detection and resolution included lack of access to patient’s/client’s medical history and lack of software for DTRP detection.

**Conclusions:**

The community pharmacists displayed fair ability in detecting and resolving DTRPs. Several barriers preventing the optimal performance of pharmacist in DTRP identification and resolution were identified including inaccessibility of patient’s/client’s medical history. The regulatory authority of pharmacy education and practice in Nigeria need to mount Continuing Education Program to address this deficit among community pharmacists.

**Supplementary Information:**

The online version contains supplementary material available at 10.1186/s12913-022-07535-z.

## Background

Inappropriate drug use, whether over-use or under-use, can cause drug therapy-related problems. Cipolle et al. defined drug therapy problem as any undesirable event experienced by a patient that involves, or is suspected to involve drug therapy, and that interferes with achieving the desired goals of therapy and requires professional judgment to resolve [1]. The occurrence of DTRP may lead to actual or potential clinical consequences [2–4]. As stated by van Mill [3], DTRP can occur when prescribing, dispensing or taking/administering medicines. Unresolved DTRPs may adversely affect the economic, clinical and humanistic outcomes of therapy and also increase morbidity and mortality rate [2]. There has been an increase in the number of hospital admissions resulting from DTRPs. According to a systematic review by Ayalew et al. [5], the prevalence of hospital admission due to DTRPs range from 1.3–43% and 7% of hospitalized patients were reported to have died from drug-related problems.

Besides prescribed drugs, patients also engage in self-medication which may not be declared to the physicians or is purchased after a hospital visit. Since most drugs for self-medication and prescription refills are obtained from the community pharmacies, community pharmacists are in a vantage position to detect and resolve DTRPs like their hospital counterpart. Pharmacists are concerned with the optimization of patient’s pharmacotherapy outcomes by employing pharmaceutical care. The pharmacist’s contribution to resolving DTRPs may be evaluated directly by assessing the patient’s clinical outcomes or indirectly by assessing the number of DTRPs identified and resolved [6]. The methods used to achieve this include prospective and retrospective reviews of prescriptions, home medicines and case notes [7–10], In another study tools were employed to detect DTRPs [11].

The interventions made by pharmacists in the identification and resolution of DTRPs and their acceptance are sometimes viewed as evidence of clinical significance [6]. Though, this may not be true in all cases as some of the interventions may lack clinical relevance [6]. The methods used in the evaluation of the number of DTRPs detected and resolved across studies vary [8, 12, 13]. To overcome this, some studies employed the use of an independent quality assessment team to evaluate pharmacists activities relating to DTRPs, albeit, retrospectively [8, 12, 14]. However, drug therapy-related problem identification and resolution are mostly self-reported and sometimes not all the interventions are documented. Thus, the reviewers or the independent quality assessment team are only able to determine the relevance or importance of the self-reported DTRPs [15]. To improve this, we considered using quality assessment team or panel of experts prospectively, that is, to predetermine the number and types of DTRPs identified and other activities of the pharmacists relating to the resolution of DTRPs. This is possible with the use of simulated patients (SPs) otherwise referred to as mystery patient, pseudo-patient, mystery shopper and standardised patient [16, 17].

Simulated patients have been used as a methodological tool for objective assessment of pharmacy practices and evaluation of the quality of pharmacists cognitive services [16–21]. These include counselling and education of patients [20], treatment of minor and major ailment [16, 17], and assessment of pharmacists public health services [21]. A SP is a trained individual who enacts a predefined, sometimes scripted, scenario or vignette in a pharmacy to assess a specific pattern of behaviour of the pharmacist or pharmacy staff. Some of the advantages of using SPs are its flexibility, adaptability, standardization, and availability of individuals and possible provision of feedback to the pharmacists [17].

This study, therefore, employed the use of SP model and an independent quality assessment team to evaluate and quantify the ability of community pharmacists to identify and resolve DTRPs presented by simulated patients vis-à-vis identification and investigation of DTRPs and recommendations for resolution. In addition, the study also evaluated the types of DTRPs frequently seen in the pharmacy and the pharmacists’ perceived barriers to the identification and resolution of DTRPs.

## Methods

### Study design and participant selection

This cross-sectional study used the SP model and questionnaire survey. The study was conducted in community pharmacies from October 2019 to January 2020 in Ibadan, a metropolitan city in southwest Nigeria and the second-largest city in the country. The city has 11 local government areas (LGAs) comprising of five urban LGAs in the city and six semi-urban LGAs in the town. The local governments are the third tiers of government in Nigeria. At the time of the study, there were 171 community pharmacies in the 11 LGAs according to the Pharmacists Council of Nigeria register. Four registered community pharmacies were targeted to be selected from each LGA. The number of community pharmacies in each of the semi-urban LGAs ranged from 1 to 22 while there were 9–24 pharmacies in each of the urban LGA. Two of the semi-urban LGAs namely Ono-Ara and Oluyole LGAs, had one and three registered community pharmacies, respectively. Based on this unequal distribution of community pharmacies in the LGAs, the four community pharmacies in Ono-Ara and Oluyole LGAs were purposively selected because of the low number of pharmacies in these LGAs. Four community pharmacies per LGA were selected from the remaining nine LGAs using computer-generated random numbers. The total number of community pharmacies selected for the study was 40.

The superintendent pharmacists in the selected community pharmacies were informed of the nature of the study which included a questionnaire guided survey, impromptu SP visits, and the recording of the conversation between the pharmacist and the SP, but the identities of the SPs and the time of visits were not revealed to the pharmacists. Consent to conduct the study was sought from each superintendent pharmacist. Forty community pharmacies were planned to be visited. Intern pharmacists and pharmacists on the National Youth Service Corp program (a post-internship mandatory one-year national service programme for fresh graduates) [16, 22] were excluded from the study. The lag time, time between the receipt of the last written informed consent from the superintendent pharmacist and the first SP visit to the first pharmacy, was 3 weeks.

### Simulated patients

Three SPs (2 males - 23 and 45 years old, and a 37-year-old female) presented the three vignettes outlined in Table 1 at the community pharmacies. The older male and the female SPs were graduates of Theatre Arts and the younger male SP was an undergraduate final year pharmacy student. Briefly, in vignette 1, a young male supposedly diagnosed with anaemia, pleurisy, duodenal ulcer, and *Tinea pedis* presented a prescription to the pharmacist and would like to know if any of the drugs could cause him any harm as he does not like taking drugs. In vignette 2, a 45-year-old known type 2 diabetes mellitus and hypertensive patient took his medications pack to the pharmacist to help identify if his medications or his diseases were responsible for his weakness. Vignette 3 described a female hypertensive and peptic ulcer patient who had uncontrolled blood pressure due to non-adherence to her hypertensive medications. She took her current prescription to the community pharmacist and sought counsel. The vignettes described in Table 1 were designed by one of the authors, SJS, based on experiences garnered from precepting students on community pharmacy postings. With the assistance of a general practice physician in a private hospital, the pseudo-prescriptions, used for the presentation of vignettes 1 and 3 by the SPs were written solely for the study.

**Table 1:** Description of vignettes re-enacted at the pharmacies by the simulated patients

**Vignette 1**		
**Background**	**Prescription/Medications**	**Medical and medication history**
The patient experienced stomach pain at night for over 2 weeks and had a fungal infection in between the toes. He claimed to have a low blood level and pain on the right side of his chest when he inhales. He had been coughing for a week. His physician diagnosed anaemia, pleurisy, duodenal ulcer, and *Tinea pedis*. He presented the following prescription to the pharmacist.	Prescription presented to the pharmacist at each pharmacy. **Name: TJB** **Gender: Male** **Age: 23 yrs** **Rx** Tab. Fluconazole 150 mg q12hr × 2/52 Tab. Ciprofloxacin 500 mg q12hr × 3/52 Tab. Omeprazole 20 mg nocte × 2/52 Tab. Ferrous gluconate 300 mg q8hr × 2/52	The simulated patient presented the prescription to the pharmacist and identified that he is the owner. He informed the pharmacists of the physician diagnoses but would like to know if any of the drugs would cause him any harm as he does not like taking medications. If the pharmacist expresses concern about the prescription and would like to speak with his physician, the simulated patient was to provide the contact number of SJS who would clarify the concern of the pharmacist. If the pharmacists refused to dispense the medications, the simulated patient must ask why? but if the pharmacist chose to dispense them the simulated patient must buy the medications. If asked of any known allergy, the SP would answer that he has no allergy.
**Vignette 2**		
**Background**	**Prescription/Medications**	**Medical and medication history**
A 45-year-old semiliterate young male took his medications to the pharmacist to help identify if his medications or his diseases were responsible for his weakness. He has type 2 diabetes and hypertension. He was feeling weak and dizzy for the last 3 days and sometimes felt like fainting. His fasting blood glucose level and blood pressure were 60 mg/dL and 126/79 mmHg, respectively that morning before coming to see the pharmacist. The following medications were what he brought to show the pharmacist.	Tab. Diamet® (Glibenclamide 5 mg) q24hr Tab. Lisinopril 10 mg q24hr Tab. Clamide® (Glibenclamide 5 mg) q24hr Syr. Coflin Linctus® 15 ml q6hr Tab. Metformin 1000 mg q12hr Tab. Diclofenac 100 mg q24hr	He asked to speak with the pharmacist only and expressed his concern. He gave his medication pack to the pharmacist and informed him of his medical conditions. If the pharmacist asked why he was taking Diamet® and Clamide®, brands of glibenclamide, together, the SP claimed that his wife bought Clamide® for him with other drugs, but he had been using Diamet®. He did not know they were the same drug. If the pharmacist asked why he was using Coflin Linctus® and ibuprofen, he responded that he had a dry cough and his knees hurt. He bought the two drugs when his friend recommended them.
**Vignette 3**		
**Background**	**Prescription/Medications**	**Medical and medication history**
A 37-year-old female took the prescription below to the pharmacy. She is a known hypertensive and gastric ulcer patient.	**Name: MN** **Gender: Female** **Age: 37 years** **Rx** Tab. Hydrochlorothiazide 25 mg q12hr × 1/12 Tab. Amlodipine 10 mg daily × 1/12 Tab, Aspirin 75 mg daily × 1/12 Tab. Omeprazole 20 mg q12hr × 1/12 Tab. Furosemide 40 mg q24hr × 1/12 Tab. Clopidogrel 75 mg q24hr × 1/12	She wanted to buy the medications but asked to speak with the pharmacist first and she informed the pharmacist that she is hypertensive and has gastric ulcer. She claim that it was her second prescription for her condition. She was diagnosed a month ago and her blood pressure at the hospital this morning was 153/92 mmHg. She told the pharmacist that she has not been taking her medications as she should and that she is willing to cooperate now that her blood pressure is not well controlled. She provided the pharmacists with other relevant information when asked, such as her allergies and that she has no other known medical condition or oedema. She wants the pharmacist to counsel her. If the pharmacist is concerned about her medications and wanted to speak with her physician, she provided the contact number of the pseudo-physician who is one of the authors (SJS). SJS addressed the concern of the pharmacist such as the withdrawal of furosemide when the patient had no oedema and the removal of Aspirin and the modification of the frequency of use of Hydrochlorothiazide if any of the pharmacists suggested these.

Calcimax® – contain Calcium carbonate, Magnesium hydroxide, Zinc sulphate and Vitamin D_3_; Coflin Lintus® – contain Chlorpheniramine maleate, Ammonium Chloride, Sodium citrate, Menthol and Ephedrine hydrochloride.

Before the commencement of the study, a 5-member quality assessment panel of experts (made up of three pharmacists in academia from the department of Clinical pharmacy and two community pharmacists) were presented with the three vignettes by the authors. The panel in turn predetermined through a minimum of 80% consensus: (a) the number and types of DTRPs in the vignettes, (b) actions the pharmacist should take to investigate the identified DTRPs, (c) appropriate recommendations the pharmacist should make. The quality assessment panel of expert used Cipolle et al. [1] classification of DTPs and Epocrates® (a point of care medical application) to select the items above for each vignette. If a drug cause more than one DTRPs, the most important was identified. Also, only drug interactions flagged by Epocrates® as “Serious” or “Use Alternative” were listed as DTRPs. A checklist was prepared based on the panel’s consensus (Supplementary Table A1) to assess the ability of the community pharmacists to identify and resolve DTRPs.

Each SP was trained by the authors through 3–5 repeated 2-h mock presentations of the vignettes prior to the re-enactment of the vignettes at the community pharmacies. Table 1 was used as a guide by the SPs on the information to provide to the pharmacist and how to respond appropriately to the pharmacists’ questions. The authors acted as pharmacists during the mock presentations and the training continued until the authors were satisfied that the SP presentations were standardized.

Subsequently, each of the three SPs made a separate impromptu visit to each pharmacy at 2 weeks intervals in sequential order. At each pharmacy, the SPs spoke with the salespersons and requested audience with the superintendent pharmacist on duty. Once the pharmacist’s attention has been obtained the SP presented either prescription or medication pack to the pharmacist and provided medical and medication history background information when asked. If the pharmacist failed to highlight any potential or actual DTRP, the SP prompted the pharmacist by asking “Is there anything I should be concerned about with this prescription or medications?” Because SPs were used, pharmacists request for clarifications from physicians were directed to one of the authors, SJS, who gave appropriate responses. The conversation between the SPs and the pharmacists was recorded and transcribed verbatim. Studies employing the simulated patient method using audio recording of conversation among community pharmacists had been conducted in the same city [16, 22]. Between 12 and 14 pharmacies were visited per week and the SPs had a week break in-between to avoid actor’s fatigue. The SPs were renumerated after the completion of the visits to the pharmacies.

From the transcripts of the conversation between the pharmacists and the SPs, the two authors independently extracted; (1) the types of DTRPs identified, (2) actions taken to investigate it and (3) the recommendations made to resolve the DTRPs identified using the checklist generated from the expert panel consensus. Differences were resolved through mutual agreement.

### Questionnaire

A self-administered structured questionnaire was given to each pharmacist and retrieved same day by the second author, LSD, after the SPs visits. The questionnaire contained 10 questions on demographic characteristics, a 19-item Likert scale on perceived barriers to DTRP detection and resolution, and another 17-item Likert scale on the types of DTRPs identified and resolved in the pharmacy in the past seven days. An 11-point bipolar scale was employed for each of the two Likert scales. For the 19-item scale, 0 = not a barrier; 10 = very strong barrier while for the 17-item scale 0 = Not seen; 10 = Seen every time. The questionnaire was developed by the authors after literature review [23–25]. The sample questionnaire can be found as Supplementary File A2.

The content and face validity were assessed by four scholars (lecturers in the Department of Clinical Pharmacy who are versatile in the design and use of questionnaire) and through pre-test among 10 non-participating community pharmacists, respectively. For the content validity, all the items considered as extremely relevant by all the scholars were included in the scales used, based on the recommendation of Lynn (1986) [26]. For the pre-test, two community pharmacies per LGA were conveniently selected from five urban LGAs. The content validity ensured that the items in the questionnaire addressed the objectives of the study while the face validity ascertained the understanding of the questions by the prospective participants. The questionnaire was retrieved from each participant after completion. The pharmacists were asked after the questionnaire-guided survey if they suspected the visit of any SPs. Two pharmacists suspected a SP visit. One was correct and the other was inaccurate.

### Data analysis

Data were presented as frequencies, percentages, mean ± standard deviation, and median (interquartile range). Each item on the checklist, mentioned by the pharmacist, was assigned a numerical value of “1” and the item not mentioned was scored “0”. Mean scores were calculated and converted to percentages. The percentage ability to identify and resolve DTRPs (a composite score from (i) the number of DTRPs identified, (ii) the actions taken to investigate the identified DTRPs, and (iii) the appropriate recommendations made) was calculated as 100(Mean score obtained)/Mean score obtainable, for each vignette. An overall percentage ability to detect and resolve DTRPs was also computed similarly. The percentage ability to detect and resolve DTRPs was further classified through the expert panel consensus as Poor ability (≤ 30%), Fair ability (> 30 - ≤ 50%), Moderate ability (> 50 - ≤ 70%) and High ability (> 70%).

The perceived barriers to DTRP detection and resolution were classified as Weak barrier (Median score, MS: 0–3), Moderately strong barrier (MS: 4–7), and Strong barrier (MS: 8–10). The DTRPs identified and resolved in the pharmacy in the past 7 days were classified as Rarely seen (MS: 0–3), Sometimes seen (MS: 4–7)-, and Often seen (MS: 8–10).

The distribution of the overall scores for percentage ability to detect and resolve DTRPs was tested for normality and homogeneity of variance. Mann-Whitney U tests was used to evaluate the association between gender and the overall percentage ability to detect and resolve DTRPs score. Kruskal-Wallis test on the other hand was used to determine if the distribution of the overall score for percentage ability to detect and resolve DTRPs was the same across the categories of age, year since graduation from pharmacy school, years of community pharmacy experience, and additional qualification. The analysis was performed using the Statistical Package for Social Sciences Windows version 25 (IBM Corp, New York, U.S.A.). Statistical significance was set at *p* < 0.05.

## Results

Forty community pharmacists who gave written informed consent were visited but only 36 (90%) completed the study and were visited by the three SPs. Two of the pharmacists were not on duty after two repeated visits by the first SP (the 23 years old male), while the other two pharmacists were known to the third SP (the 35 years old female). A total number of 108 visits were made to the selected pharmacies by the three SPs. The mean age of the pharmacists was 29.53 ± 5.20 years (Table 2), with males being predominant 21 (58.3%). About 22 (61.2%) of the pharmacists had 3 years and above community pharmacy practice experience.

**Table 2:** Demographic characteristics of community pharmacists

Demographic characteristics	Mean ± SD	Frequency (%)
**Gender**		
Male		21 (58.3)
Female		15 (41.7)
**Age, years**	29.53 ± 5.20	
**≤** 27		14 (38.9)
28–29		9 (25.0)
30+		13 (36.1)
**Year since graduation**	5.12 ± 2.15	
≤ 4		15 (41.7)
5–6		12 (33.3)
7+		9 (25.0)
**Years of community pharmacy practice**	3.83 ± 2.66	
≤ 2		14 (38.9)
3–4		11 (30.6)
5+		11 (30.6)
**Highest academic qualification**		
B.Pharm		30 (83.3)
PharmD^a^		3 (8.3)
M.Sc.		3 (8.3)

*B.Pharm* Bachelor of Pharmacy is required for pharmacy practice in Nigeria, ^a^*PharmD* Doctor of Pharmacy is acquired after an intensive 1-year program for B. Pharm degree holders, *M. Sc* Master of Science.

Based on the outcome of the validity assessments of the questionnaire, two questions in the Likert scale were reconstructed. Too much workload was replaced with excess workload, and lack of access to patient’s case file was replaced with lack of access to patient/client medical history. More subcategories of DTRPs on adherence were added to the Likert scale evaluating pharmacists encounter with DTRPs in the last 7 days. These included patients cannot afford drug product, directions on the prescription not understood, and patient prefers not to take the prescribed medications. Additional options in a few of the sociodemographic variable were provided, such as the inclusion of Pharm. D as the third option for the variable Highest academic qualification. The questions on years of graduation and practice experience were change to continuous variable (participants were required to fill in the appropriate number of years) instead of categorical variable (range of options for participants to choose from). The Cronbach alpha coefficient for the two scales in the questionnaire was 0.862 and 0.915.

### Vignette 1: 23-year-old SP with prescription for anaemia, pleurisy, duodenal ulcer, and *Tinea pedis*

The pharmacists identified 1.58 out of the possible 3 DTRPs on the prescription. Twenty-eight (77.8%) and 25 (69.4%) pharmacists identified the dose of fluconazole and ciprofloxacin given to the patient as high, respectively. None of the pharmacists checked for drug interactions, but the majority 24 (66.7%) requested to make some clarification from the physician. Most of the pharmacists recommended a reduction in the dose of fluconazole and duration of treatment with ciprofloxacin (Table 3).

**Table 3:** DTRPs identified and resolved by pharmacists in a 23-year-old SP with a prescription

Classification and types of DTRPs	Identified n (%)	Not identified n (%)
**Dosage too high (*Prescribed dose too high*)**		
Fluconazole 150 mg instead of 50 mg daily or 150 mg weekly for *Tinea pedis*	28 (77.8)	8 (22.2)
**Dosage too high (*Duration of treatment too long*)**		
Ciprofloxacin 500 mg twice daily for 3 weeks instead of 500 mg twice daily for 7- days for the treatment of pleurisy	25 (69.4)	11 (30.6)
**Dosage too low (*Drug interaction*)**		
Omeprazole + ferrous gluconate (omeprazole decreases the level or effect of ferrous gluconate)^a^	4 (11.1)	32 (88.9)
**Action taken to investigate DTRPs**	**Action taken** **n (%)**	**Action not taken** **n (%)**
Checked for drug interactions	0 (0.0)	36 (100.0)
Inquire or make clarification from the prescriber	24 (66.7)	12 (33.3)
**Recommendations made to resolve the DTRPs**	**Recommendation made** **n (%)**	**Recommendation not made** **n (%)**
Recommended that prescriber reduce the duration of therapy of ciprofloxacin tablets for pleurisy to 7 days.	23 (63.9)	13 (36.1)
Recommended the reduction of fluconazole dosage to 50 mg daily.	22 (61.1)	14 (38.9)

*DTRPs* Drug therapy-related problems, *n* number of pharmacists, ^a^Serious - Avoid or Use alternative, *SP* simulated patient.

### Vignette 2: 45-year-old SP who had type 2 diabetes and hypertension

Most of the pharmacists 31 (86.1%) were able to detect that the SP was taking a high dose of glibenclamide (two brands of the same product taken together). Thirty-two pharmacists (88.9%) suggested the stoppage of one of the glibenclamide brands (Table 4). Likewise, 32 (88.9%) pharmacists asked the SPs why he was taken the medicines he brought to the pharmacy. Few of the pharmacists, 3 (8.3%), identified a potential adverse drug reaction with the use of diclofenac and lisinopril together, which may result in reduced renal function.

**Table 4:** DTRPs identified and resolved by pharmacists in a 45-year-old type 2 diabetes and hypertensive SP.

Classification and types of DTRPs	Identified n (%)	Not identified n (%)
***Drug therapy is used for an avoidable adverse drug reaction/side effects associated with another medication***		
Coflin Lintus® possibly for cough associated with lisinopril.	4 (11.1)	32 (88.9)
**Adverse drug reaction (*A drug interaction causes an undesirable reaction that is not dose-related*)**		
Diclofenac + lisinopril decreases renal function^a^	3 (8.3)	33 (91.7)
**Dose too high (*The dose too high for the patient*)**		
Glibenclamide 10 mg taken twice daily because of duplicate product.	31 (86.1)	5 (13.9)
**Action taken to investigate DRP**	**Action taken** **n (%)**	**Action not taken** **n (%)**
Checked for drug interaction.	2 (5.6)	34 (94.4)
Asked for clarification concerning the medicines.	32 (88.9)	4 (11.1)
Probe the patient further about other symptoms of hypoglycaemia e.g., tremor, increased heart rate	21 (58.3)	15 (41.7)
**Recommendations made to resolve the DTRPs**	**Recommendation made** **n (%)**	**Recommendation not made** **n (%)**
Suggested the stoppage of diclofenac tablets.	6 (16.7)	30 (83.3)
Suggested the discontinuation of one of the glibenclamide brand.	32 (88.9)	4 (11.1)
Suggested the patient see the physician for a possible replacement for lisinopril since the patient experienced episodes of uncomfortable dry cough.	4 (11.1)	32 (88.9)
Suggested the discontinuation of Coflin Lintus®.	3 (8.3)	33 (91.7)

*DTRPs* Drug therapy-related problems, *n* number of pharmacists, ^a^Serious - Avoid or Use alternative, *SP* simulated patient.

Coflin Lintus® – contain Chlorpheniramine maleate, Ammonium Chloride, Sodium citrate, Menthol and Ephedrine hydrochloride).

### Vignette 3: 37-year-old SP who was a known hypertensive and gastric ulcer patient

Unnecessary use of clopidogrel with aspirin and frusemide with hydrochlorothiazide was identified by 25 (69.4%) and 16 (44.4%) pharmacists, respectively. Other identified DTRPs in vignette 3 are also listed in Table 5. Thirteen (36.1%) and 17 (47.2%) community pharmacists, respectively made some clarification from the SP, and the patients’ physician (Table 5). Twenty-six (72.2%) pharmacists suggested the discontinuation of clopidogrel while a few 12 (33.3%) suggested a reduction in the frequency of use of hydrochlorothiazide to once daily.

**Table 5:** DTRPs identified and resolved by pharmacists in a 37-year-old hypertensive SP with gastric ulcer

Classification and types of DTRPs	Identified N (%)	Not identified N (%)
**Unnecessary drug therapy (*Multiple drug products were prescribed when single drug therapy is required*)**		
Clopidogrel + aspirin	25 (69.4)	11 (30.6)
Frusemide + hydrochlorothiazide	16 (44.4)	20 (55.6)
**Unnecessary drug therapy (*No valid medication indication for drug at this time)***		
Frusemide is not indicated since there was no oedema.	15 (41.7)	21 (58.3)
**Dosage too low (*A drug interaction reduces the amount of drug available*)**		
Omeprazole decreases the level of clopidogrel^a^	2 (5.6)	34 (94.4)
**Dose too high (*The dose too high for the patient*)**		
Hydrochlorothiazide 25 mg twice daily	6 (16.7)	30 (83.3)
**Action taken to investigate DTRP**	**Action taken** **n (%)**	**Action not taken** **n (%)**
Checked for drug interactions.	0 (0.0)	36 (100.0)
Asked the simulated patient for clarification on medication-related issues.	13 (36.1)	23 (63.9)
Made clarification from the prescriber on the use of aspirin and clopidogrel together.	17 (47.2)	19 (52.8)
Made clarification from the prescriber on the concomitant use of Hydrochlorothiazide and furosemide	17 (47.2)	19 (52.8)
**Recommendations made to resolve the DTRPs**	**Recommendation made** **n (%)**	**Recommendation not made** **n (%)**
Suggested to the physician the use of hydrochlorothiazide only instead of hydrochlorothiazide and frusemide together.	14 (38.9)	22 (61.1)
Suggested the discontinuation of clopidogrel	26 (72.2)	10 (27.8)
Suggested reduction in hydrochlorothiazide 25 mg frequency of use to once daily.	12 (33.3)	24 (66.7)

*DTRPs* Drug therapy-related problems, ^a^Serious - Avoid or Use alternative, *SP* simulated patient.

In total, 4.42/11 (40.2%) DTRPs were identified, 3.50/9 (38.9%) actions were taken to investigate the DTRPs, and 3.94/9 (43.8%) recommendations were made to resolve identified DTRPs (Table 6). The percentage ability of the community pharmacists to detect and resolve DTRPs varied slightly from one vignette to another (vignette 1–49.3%, vignette 2–39.1%, vignette 3–38.8%). But overall, the percentage ability of the community pharmacists to detect and resolve DTRP was fair (40.9%). The most frequently encountered DTRP in the community pharmacies was the problem of adherence where patients could not afford the drugs (Table 7). Part of the strong barriers to the detection and resolution of DTRPs as reported by the community pharmacists were impatience on the part of patients/clients, and lack of access to patient’s/client’s medical history (Table 8).

**Table 6:** Mean score with the percentage ability to detect and resolve DTRPs

Description	Mean score ± S.D	Maximum score obtainable	% Ability to detect and resolve DTRPs, (95% CI)	Interpretation
**Vignette 1**				
DTRPs identified	1.58 ± 0.97	3		
Actions taken to investigate DTRPs	0.67 ± 0.48	2		
Recommendations made	1.25 ± 0.94	2		
**Composite score**	**3.50 ± 2.26**	**9**	**49.5 (38.5, 60.6)**	**Fair**
**Vignette 2**				
DTRPs identified	1.06 ± 0.48	3		
Actions taken to investigate DTRPs	1.53 ± 0.61	3		
Recommendations made	1.25 ± 0.73	4		
**Composite score**	**3.83 ± 1.18**	**10**	**39.1 (35.2, 43.2)**	**Fair**
**Vignette 3**				
DTRPs identified	1.78 ± 1.46	5		
Actions taken to investigate DTRPs	1.31 ± 1.26	4		
Recommendations made	1.44 ± 1.16	3		
**Composite score**	**4.53 ± 3.54**	**12**	**38.8 (28.6, 49.0)**	**Fair**
Total DTRPs identified	4.42 ± 1.90			
Total action taken to investigate the DTRPs	3.50 ± 1.61			
Total Recommendations made	3.94 ± 1.66			
**Overall composite score**	**11.86 ± 4.70**	**29**	**41.0 (35.5, 46.4)**	**Fair**

*CI* Confidence Interval, *S.D* Standard Deviation.

% Ability to detect and resolve DTRPs, (a composite score of the percentage ability to detect DTRPs, investigate it, and make appropriate recommendation) for each vignette and the three vignettes combined:

Poor ability (≤ 30%).

Fair ability (> 30 - ≤ 50%),

Moderate ability (> 50 - ≤ 70%) and.

High ability (> 70%).

**Table 7:** Types of DTRPs seen in the pharmacies in the last 7 days by community pharmacists

DTRP Categories	Drug therapy-related problems	Description of encounter Median (IQR)
		**Encounter sometimes seen**
Need Additional Therapy	Untreated condition	6.00 (3.25–7.00)
Unnecessary Drug Therapy	Non-drug therapy should have been recommended	6.50 (4.00–7.00)
	Duplicate therapy	6.00 (2.00–8.75)
Ineffective Drug	Dosage form prescribed is inappropriate	6.50 (3.00–9.00)
	Contraindication present	5.00 (0.25–8.00)
	Drug prescribed will not be effective for the patient	5.00 (2.00–7.00)
Dose too Low/Dose too High	Wrong dose prescribed	7.00 (2.00–8.00)
Dose too Low	Frequency of use too long	7.00 (3.00–8.00)
	Duration of therapy too short	5.50 (1.00–7.00)
Adverse Drug Reaction	Drug interaction present	6.00 (2.00–7.00)
	Drug prescribed may not be safe for patient	6.00 (4.00–7.00)
	Possibility of patient experiencing ADR	5.00 (1.00–7.00)
Adherence	Prescribed product not readily available	6.00 (2.50–7.25)
	Patient cannot tolerate the prescribed dosage form	4.00 (1.25–7.00)
	Directions on the prescription not understood	5.00 (0.00–8.00)
		**Encounter often seen**
	Patient cannot afford drug product	8.00 (6.25–10.00)
	Patient prefers not to take the prescribed medications	**Encounter rarely seen** 3.00 (1.00–8.00)

*DTRPs* Drug therapy-related problems.

Description of DTRP encounters based on Median score:

0–3: Rarely seen,

4–7: Sometimes seen,

8–10: Often seen.

**Table 8:** Community pharmacists’ perceived barriers to drug therapy-related problems detection and resolution

Barriers to DTRP detection and resolution	Categories of barriers to DRP Median (IQR)
**Patient-related barriers**	
	**Strong barriers**
Impatience on the part of patients/clients	8.00 (5.00–10.00)
Lack of access to patient/client medical history	8.00 (6.00–9.00)
	**Moderately strong barriers**
Patient’s level of education	7.00 (6.00–8.00)
Patient’s attitude	7.00 (5.00–8.00)
**Interprofessional collaboration barriers**	
Difficulty in contacting the physician	6.50 (4.25–9.00)
Negative physician attitude towards pharmacist recommendation	6.50 (5.00–9.00)
**Practice/profession-related barriers**	
Excessive workload	6.50 (5.00–8.75)
Lack of adequate training	6.50 (3.25–8.75)
Lack of time	5.00 (4.00–8.00)
Inadequate qualified personnel	6.00 (3.00–7.00)
Lack of remuneration for pharmacist	5.50 (3.25–8.00)
Lack of documentation skill	6.00 (5.00–8.75)
Inadequate communication skill with patients	4.50 (2.25–8.00)
Difficulty in accessing drug information	5.00 (2.25–7.75)
**Pharmacy-related barriers**	
Lack of software to make the detection of DTRPs easy	5.50 (5.00–9.00)
Lack of space	4.00 (0.25–5.75)
Lack of motivation for pharmacist	4.50 (1.00–8.00)
Lack of internet facility	4.50 (1.25–6.75)
Pharmacy layout	5.00 (1.00–6.00)

*DTRPs* Drug therapy-related problems, *IQR* Interquartile range,

Categories of barriers:

Median score 0–3: Weak barrier,

Median score 4–7: Moderately strong barrier,

Median score 8–10: Strong barrier,

A Mann-Whitney U test revealed no significant difference in the overall percentage ability to detect and resolve DTRPs of males (Mean rank = 20.14, *n* = 21) and females (Mean rank = 16.20, *n* = 15), *U* = 123, *p* = 0.279. Kruskal-Wallis test showed no significant differences in the categories of age, year since graduation, and years of community pharmacy practice experience for the overall percentage ability to detect and resolve DTRPs (*p* > 0.05), Table 9. Kruskal Wallis test also revealed a statistically significant difference in the overall percentage ability to detect and resolve DTRPs across the three groups of pharmacists’ highest academic qualifications (B.Pharm, *n* = 30, Mean rank = 17.25; Pharm. D, *n* = 3, Mean rank = 34.83; M.Sc.,. *n* = 3, Mean rank = 14.67), χ^2^ (2, *n* = 36) = 8.043, *p* = 0.018, Table 9. Pharmacists with Pharm. D as additional qualification had the highest mean rank.

**Table 9:** Association between demographic variables and overall percentage ability score to detect and resolve drug therapy-related problems

Demographic characteristics	N	Mean rank	***p***-value
**Gender**			
Male	21	20.14	0.279^a^
Female	15	16.20	
**Age, years**			
**≤** 27	14	19.29	
28–29	9	16.39	0.785^b^
30+	13	19.12	
**Year since graduation**			
≤ 4	15	15.23	
5–6	12	22.33	0.218^b^
7+	9	18.83	
**Years of community pharmacy practice**			
≤ 2	14	16.68	
3–4	11	19.23	0.697^b^
5+	11	20.09	
**Highest academic qualification**			
B.Pharm	30	17.25	
PharmD	3	34.83	0.018*^b^
M.Sc.	3	14.67	

*B.Pharm* Bachelor of Pharmacy is required for pharmacy practice in Nigeria, *PharmD* Doctor of Pharmacy.

*P < 0.05, ^a^Mann Whitney U test, ^b^Kruskal Wallis test

## Discussion

The community pharmacists in this study displayed a fair ability to detect and resolve DTRPs, vis-à-vis identification of DTRPs, investigation of the causes and recommendations for resolution. The DTRPs mostly identified were dosage too high and unnecessary drug therapy. Others were the need for additional drug therapy and adverse drug reactions. Interestingly, most of the pharmacists did not check for drug interactions. However, a greater number of the pharmacists asked the SPs for further clarifications on medication-related issues and between 47 and 67% of the pharmacists contacted “the physicians” of the SPs on suspected DTRPs and suggested recommendations on how to resolve identified DTRPs. As high as 89% of the pharmacists made appropriate recommendations which bordered on dosage adjustment, drug discontinuation, and drug substitution.

The fair ability to detect and resolve DTRPs (41%) displayed by the community pharmacists differs from two other similar studies where independent quality assessment teams were also used to evaluate pharmacists’ DTRP activities. In Ewan and Greene [15] and Gisev et al. [14], the expert review panels deemed 91 and 76% of the DTRP interventions made by the pharmacists appropriate, respectively. In these two studies, the findings of the pharmacists were retrospectively assessed by experts while the DTRPs in this study was predetermined by the panel of experts and compared with the pharmacist’s findings. Interstudy comparison may be difficult as studies use different scoring criteria [6, 11, 27–33], such as the use of an independent assessment team or panel, to assess the quality of pharmacists DTRPs interventions. The composition of such panel, which vary across studies, may impact on the judgment or final evaluation of the performance of the pharmacists, since members of the panel may have different concept of clinical relevance of DTRP interventions [8, 12, 14]. Nevertheless, the ability of community pharmacist in this study to detect and resolve DTRPs was low compared with the two studies. The differences may be due in part to the study settings, study populations, definition of DTRPs, and the methods of identifying the DTRPs [5, 34] but mostly as a result of the study design. To the best of our knowledge, this is the first time a SP model along with an independent quality assessment panel of experts who determined the DTRPs and possible resolutions in a given scenario prospectively was used to assess pharmacist’s ability to identify and resolve DTRPs. Several other studies used the independent quality assessment panel of experts to determine the quality of identified and resolved DTRPs by pharmacists retrospectively [8, 12, 14].

The DTRPs, dosage too high and unnecessary drugs, were identified by most of the pharmacists compared with a study in Ethiopia reporting a low level (4%) of identification of the DTRP - dosage too high [7]. In two other related studies conducted in Minnesota, U.S.A and Jos Nigeria, the lowest-rated DTRP was unnecessary drug therapy [32, 35]. But in other studies, DTRPs such as drug interactions, unnecessary drugs therapy, and adverse drug reaction, identified by few community pharmacists in this study, were commonly reported among patients [7, 32, 36]. The reason why few of the pharmacists did not identify some of these frequently reported DTRPs could be because the DTRPs were infrequently seen in the pharmacy (Table 7).

The fair ability displayed by the pharmacists to detect and resolve DTRPs may also be due to some of the perceived barriers mentioned by the pharmacists. These included inadequate training and lack of documentation skill among others. This is corroborated by Williams et al. [37] study which reported a strong correlation between pharmacists clinical knowledge and level of additional training, and the ability of pharmacists to detect, obtain relevant information and proffer resolution for DTRPs. The finding in this study agrees with Williams et al. [37] report because community pharmacists with Pharm. D degree seem to perform better in detecting and resolving DTRPs.

In Nigeria, Pharm. D may be acquired after an intensive one-year clinically oriented program for Bachelor of Pharmacy degree holders [38]. The National University Commission approved the Pharm. D degree program as undergraduate National degree in 2016 [39, 40]. Currently the Bachelor of Pharmacy degree is the minimum requirement to practise as pharmacist in Nigeria but soon the Pharmacists Council of Nigeria may set the Pharm. D degree as the minimum requirement [41]. Presently 11 Universities have been approved to run the Pharm. D degree programme [42].

It should be taken into consideration the diversity of therapeutic areas covered in the vignettes and the number of issues within a prescription that might have impacted the results not under-estimating the need for pharmacists to have identified the DTRPs. Variations in pharmacy staff and pharmacist’s behaviour, when presented with different scenarios, have been reported in pharmacy practice [43–47]. Inconsistencies in pharmacist’s behaviour between or within vignettes could be a pointer to the underlying process that drives pharmacy practice. Such factors include lack of time, patient’s attitude, difficulty in contacting physicians, lack of remuneration or motivation among other perceived barriers to identify and resolve DTRPs as reported here and highlighted in the literature [23–25, 48–50]. The common conclusion when the pharmacist’s performance is substandard is the need for additional training. However, this may not automatically improve the quality of performance. Though, the variability in the performance of the community pharmacists reported here may be reflective of deficiencies in tailored clinical training programs. From the foregoing, there is a need for the inclusion of courses on the detection and resolution of DTRPs in Continuing Education Program for pharmacists, especially those in this study, to improve performance. However, these courses should be extended beyond the clinical perspective to include communication skills for effective interaction with patients or pharmacy clients [47]..

The inability of pharmacists to detect and resolve drug interactions may lead to the development of ADRs and subsequent hospitalization. Yet few of the pharmacists were able to spot a significant drug interaction such as the use of omeprazole with Ferrous gluconate. None of the pharmacists used Medscape, Epocrates or any drug interaction textbook. The lack of software for DTRP detection, lack of stable internet facility, and difficulty in accessing drug information were barriers perceived by the community pharmacists that could have limited the pharmacist’s ability to investigate suspected DTRPs. Hence the provision of subsidized commercially available medication review software and electronic data system by pharmacy corporate bodies might assist the pharmacists in detecting and resolving DTRPs.

From the results, it was clear that vignettes with prescriptions generated more contact with the physician and 61–89% of the pharmacists made appropriate recommendations. In a related study in Sydney by Gisev et al. [14] among clients of community mental health teams, 81% of the recommendations made by pharmacists to resolve DTRPs were judged appropriate. This high level of appropriate recommendations is in keeping with part of our findings. Pharmacists recommendations of possible resolutions to DTRPs have varied acceptance rates among physicians [8]. Because of the design of this study, we did not evaluate the level of acceptance of the pharmacist recommendations since a SP model was used and one of the authors acted as “the pseudo-physician” to receive the pharmacists call on clarifications and suggestions based on the SP’s vignette.

The national gender distribution of community pharmacists is in contrast with the global trend of female pharmacists been more predominant [41, 51, 52]. The ratio of male:female pharmacists in the country is 1.6:1. This is the same with the regional gender distribution of pharmacists. In our study male to female ratio is 1.4:1 which is almost similar to the national figure and gender distribution at the state level.

### Strengths and limitations of the study

Since most DTRPs are self-reported and subjective with its attendant limitations of honesty of reporting and recall bias [15], the design of this study afforded an objective measure of community pharmacists practice of identifying and resolving DTRPs. The study is however not without some limitations. The simulated patient model may be prone to Hawthorne effect, but the possibility was reduced using three different SPs and vignettes. The pharmacists could not have altered their behaviour during the SPs’ visits since they were unaware of the time of the visits. Only one pharmacist correctly suspected a SP visit. A limited number of SPs were used compared with the number of patients seen in the community pharmacies. However, the use of many SPs may also make the pharmacists suspicious and hence modify their behaviour. The three vignettes used do not represent the full remit of pharmacy practice and one visit per SP does not necessarily imply that the behaviour is always similar. Also, the number of community pharmacies involved in the study was small, but this was improved upon by the number of visits to the pharmacies. Furthermore, because the study was carried out in one state in Nigeria, it may not be representative of the practice of community pharmacists in other states in the country. Recall bias was also prevented by audiotaping the conversation between the pharmacists and the SPs.

## Conclusion

The community pharmacists displayed a fair ability to detect and resolve drug therapy-related problems vis-à-vis identification of drug therapy-related problems, investigation of the causes and recommendations for resolution. The findings here are limited to the vignettes used. Further studies employing a wide array of vignettes may provide more information on the ability of community pharmacists to detect and resolve drug therapy-related problems. Nonetheless, there is a need for the Pharmacists Council of Nigeria to include courses on the identification and resolution of drug therapy-related problems in Continuing Education Program for community pharmacists to enhance the practice.

## Supplementary Information

**Additional file 1.**

**Additional file 2.**

## Data Availability

The datasets generated during and/or analysed during the current study are available from the corresponding author on reasonable request.

## Abbreviations

DTRPs: Drug therapy-related problems
SPs: Simulated patients
LGAs: Local government areas
