# Supplementary material for: Drug therapy-related problem management in Nigeria community pharmacy – process evaluation with simulated patient
Source: BMC Health Serv Res. 2022 Feb 16;22:209. doi: 10.1186/s12913-022-07535-z (PMC8848586; doi:10.1186/s12913-022-07535-z)
Supplement: Supplementary file 1 — Additional file 1. [file 12913_2022_7535_MOESM1_ESM.docx]

**Supplementary Table A1: Checklist for the identification and resolution of drug therapy problems**

| **Checklist of possible DTPs, action to take to investigate it and recommendations to make** | | | |
| --- | --- | --- | --- |
| A | **Vignette 1: A 23-year-old simulated patient with prescription for anaemia, pleurisy, duodenal ulcer, and *Tinea pedis*** | | |
|  | **DTPs identified** | **DTP identified** | **DTP not identified** |
| 1 | Did the pharmacists detect that the dose of Fluconazole for the indication was too high?  (Fluconazole 150 mg instead of 50 mg daily or 150 mg weekly for *Tinea pedis*) |  |  |
| 2 | Did the pharmacist identify that the duration of treatment of pleurisy using Ciprofloxacin was too long?  (Ciprofloxacin 500 mg twice daily for 3 weeks instead of 500 mg twice daily for 7- days for the treatment of pleurisy) |  |  |
| 3 | Did the pharmacist identify that the concurrent use of Omeprazole and Ferrous gluconate may reduce the effect of the Iron preparation?  (Omeprazole decreases the level or effect of ferrous gluconate)* |  |  |
|  | **Actions taken to investigate DTPs** | **Action taken** | **Action not taken** |
| 4 | Did the pharmacist check for any drug interactions? |  |  |
| 5 | Did the pharmacist inquire or make clarification from the prescriber? |  |  |
|  | **Recommendations made to resolve the DTPs** | **Recommendation made** | **Recommendation not made** |
| 6 | Did the pharmacist recommend that the prescriber reduce the duration of therapy of Ciprofloxacin tablets for pleurisy to 7 days? |  |  |
| 7 | Did the pharmacist recommend the reduction of fluconazole dosage to 50 mg daily |  |  |
| B | **Vignette 2: A 45-year-old simulated patient who had type 2 diabetes and hypertension.** | | |
|  | **DTP identified** | **DTP identified** | **DTP not identified** |
| 1 | Did the pharmacist identify that a drug was used for an avoidable adverse drug reaction/side effects associated with another medication?  (Coflin Lintus® possibly for cough associated with Lisinopril) |  |  |
| 2 | Did the pharmacists identify that a drug interaction may cause an undesirable reaction that is not dose-related?  (Diclofenac + Lisinopril decreases renal function) * |  |  |
| 3 | Did the pharmacist identify that the dose of Glibenclamide used by the patient was too high?  (Glibenclamide 10 mg taken twice daily because of duplicate products) |  |  |
|  | **Action taken to investigate DTPs** | **Action taken** | **Action not taken** |
| 4 | Did the pharmacist check for drug interactions? |  |  |
| 5 | Did the pharmacist ask the patient for clarification concerning the other medications? |  |  |
| 6 | Did the pharmacist probe the patient further about other symptoms of hypoglycaemia e.g., tremor, increased heart rate? |  |  |
|  | **Recommendations made to resolve the DTPs** | **Recommendation made** | **Recommendation not made** |
| 7 | Did the pharmacist suggest the stoppage of diclofenac tablets.? |  |  |
| 8 | Did the pharmacist suggest the discontinuation of one of the Glibenclamide brand? |  |  |
| 9 | Did the pharmacist suggest the patient see the physician for a possible replacement for Lisinopril since the patient experienced episodes of uncomfortable dry cough? |  |  |
| 10 | Did the pharmacist suggest the discontinuation of Coflin Lintus®? |  |  |
| C | **Vignette 3: A 37-year-old simulated patient who was a known hypertensive and gastric ulcer patient.** | | |
|  | **DTP identified** | **DTP identified** | **DTP not identified** |
| 1 | Did the pharmacist identify that multiple drug products prescribed may be used when single drug therapy is required?  {Clopidogrel and Aspirin) |  |  |
| 2 | Did the pharmacist identify that multiple drug products prescribed may be used when single drug therapy is required?  (Frusemide and Hydrochlorothiazide) |  |  |
| 3 | Did the pharmacist identify that there was no valid indication for a drug at this time?  (Frusemide is not indicated since there was no oedema). |  |  |
| 4 | Did the pharmacist identify that drug interaction may reduce the amount of a drug available for activity?  (Omeprazole decreases the level of Clopidogrel) * |  |  |
| 5 | Did the pharmacist identify that the dose of a drug was too high?  (Hydrochlorothiazide 25 mg twice daily) |  |  |
|  | **Action taken to investigate DTPs** |  |  |
| 6 | Did the pharmacist check for drug interaction? |  |  |
| 7 | Did the pharmacist ask the simulated patient for clarification on medication-related issues? |  |  |
| 8 | Did the pharmacist clarify from the prescriber on the use of Aspirin and Clopidogrel together? |  |  |
| 9 | Did the pharmacist clarify from the prescriber on the use of Hydrochlorothiazide and Furosemide together? |  |  |
|  | **Recommendations made to resolve the DTPs** | **Recommendation made** | **Recommendation not made** |
| 10 | Did the pharmacist suggest to the physician the use of hydrochlorothiazide only instead of hydrochlorothiazide and frusemide together? |  |  |
| 11 | Did the pharmacist suggest the discontinuation of Clopidogrel to the physician? |  |  |
| 12 | Did the pharmacist suggest a reduction in hydrochlorothiazide 25mg frequency of use to once daily? |  |  |

DTPs – Drug therapy problems,

*Serious - Avoid or Use alternative

Coflin Lintus^®^ – contain Chlorpheniramine maleate, Ammonium Chloride, Sodium citrate, Menthol and Ephedrine hydrochloride).
