# Supplementary material for: Drug therapy-related problem management in Nigeria community pharmacy – process evaluation with simulated patient
Source: BMC Health Serv Res. 2022 Feb 16;22:209. doi: 10.1186/s12913-022-07535-z (PMC8848586; doi:10.1186/s12913-022-07535-z)
Supplement: Supplementary file 2 — Additional file 2. [file 12913_2022_7535_MOESM2_ESM.docx]

**DEPARTMENT OF CLINICAL PHARMACY**

**FACULTY OF PHARMACY**

**UNIVERSITY OF IBADAN**

**Questionnaire investigating barriers to identification and resolution of drug therapy problems**

**Dear respondent,**

Thank you for taking time out to fill this questionnaire.as the topic specified, we want to find out the different types of barriers faced by pharmacists which sometimes makes it difficult for them to detect drug therapy problems and proffer solutions to them. For clarification, A drug-related problem (DRP) is commonly defined as ‘‘an event or circumstance involving drug treatment that actually or potentially interferes with the patient experiencing an optimum outcome of medical care’’

Thank you for your time.

**Participants demographics**

**Gender:** Male [ ] Female [ ]; **Age:** _____; **Religion:** Islam [ ] Christianity [ ], Traditional [ ]

**Year of graduation:** ______; **Highest academic qualification**: B.Pharm [ ], PharmD [ ], M.Sc [ ], FPCPharm [ ]

**Years of community pharmacy experience: __________**

Kindly grade the underlisted barriers to drug therapy problems detection and resolution based on what you considered the strength of the barrier or how weighty the barrier is. If it’s not a barrier please tick **0**, but if you feel it’s a very strong barrier, please tick **10**. Please tick any number between **0** and **10** to rate each factor.

| **S/N** | **Barriers DRP detection & resolution*** | **Not a**  **barrier** |  | | | | | | | | | **Very**  **strong**  **barrier** |
| --- | --- | --- | --- | --- | --- | --- | --- | --- | --- | --- | --- | --- |
|  |  | **0** | **1** | **2** | **3** | **4** | **5** | **6** | **7** | **8** | **9** | **10** |
| 1 | Lack of time |  |  |  |  |  |  |  |  |  |  |  |
| 2 | Lack of adequate training |  |  |  |  |  |  |  |  |  |  |  |
| 3 | Inadequate communication skill with patients |  |  |  |  |  |  |  |  |  |  |  |
| 4 | Inadequate qualified personnel |  |  |  |  |  |  |  |  |  |  |  |
| 5 | Lack of space |  |  |  |  |  |  |  |  |  |  |  |
| 6 | Lack of motivation for pharmacists |  |  |  |  |  |  |  |  |  |  |  |
| 7 | Lack of renumeration for pharmacists |  |  |  |  |  |  |  |  |  |  |  |
| 8 | Impatience on the part of client/patient |  |  |  |  |  |  |  |  |  |  |  |
| 9 | Lack of software to make the detection of DRP easy |  |  |  |  |  |  |  |  |  |  |  |
| 10 | Lack of documentation skill |  |  |  |  |  |  |  |  |  |  |  |
| 11 | Lack of internet facility |  |  |  |  |  |  |  |  |  |  |  |
| 12 | Difficulty in accessing drug information |  |  |  |  |  |  |  |  |  |  |  |
| 13 | Lack of access to patient/client medical history |  |  |  |  |  |  |  |  |  |  |  |
| 14 | Patients level of education |  |  |  |  |  |  |  |  |  |  |  |
| 15 | Excessive workload |  |  |  |  |  |  |  |  |  |  |  |
| 16 | Patients attitude |  |  |  |  |  |  |  |  |  |  |  |
| 17 | Pharmacy layout |  |  |  |  |  |  |  |  |  |  |  |
| 18 | Difficulty in contacting physician |  |  |  |  |  |  |  |  |  |  |  |
| 19 | Negative physician attitude towards pharmacists’ recommendations |  |  |  |  |  |  |  |  |  |  |  |
| **Please list other barriers not included above and rate them here:** | | | | | | | | | | | | |
| 20 |  |  |  |  |  |  |  |  |  |  |  |  |
| 21 |  |  |  |  |  |  |  |  |  |  |  |  |
| 22 |  |  |  |  |  |  |  |  |  |  |  |  |
| 23 |  |  |  |  |  |  |  |  |  |  |  |  |
| 24 |  |  |  |  |  |  |  |  |  |  |  |  |

**KINDLY TURN TO THE NEXT AND LAST PAGE.**

Which of the following drug therapy problems did you encounter in the last 7-days on prescriptions that came to the pharmacy?

Please chose any number from 0 to 10 depending on how often you came across these drug therapy problems in the last 7-days. The number 0 means you did not see a prescription with this DTP while number 10 means you always see it. Numbers 1 to 9 represent other frequencies depending on how frequent you see the type of DTPs.

| **S/N** | **Drug therapy problems seen on prescription in the pharmacy** | **Never seen** |  | | | | | | | | | **Seen every time** |
| --- | --- | --- | --- | --- | --- | --- | --- | --- | --- | --- | --- | --- |
|  |  | **0** | **1** | **2** | **3** | **4** | **5** | **6** | **7** | **8** | **9** | **10** |
| 1 | Untreated conditions |  |  |  |  |  |  |  |  |  |  |  |
| 2 | Non drug therapy should have been recommended |  |  |  |  |  |  |  |  |  |  |  |
| 3 | Duplicate therapy |  |  |  |  |  |  |  |  |  |  |  |
| 4 | Dosage form prescribed is inappropriate |  |  |  |  |  |  |  |  |  |  |  |
| 5 | Contraindication present |  |  |  |  |  |  |  |  |  |  |  |
| 6 | Drug prescribed will not be effective for the patient |  |  |  |  |  |  |  |  |  |  |  |
| 7 | Wrong dose prescribed |  |  |  |  |  |  |  |  |  |  |  |
| 8 | Frequency of use too long |  |  |  |  |  |  |  |  |  |  |  |
| 9 | Duration of therapy too short |  |  |  |  |  |  |  |  |  |  |  |
| 10 | Drug interaction present |  |  |  |  |  |  |  |  |  |  |  |
| 11 | Drug prescribed may not be safe for patient |  |  |  |  |  |  |  |  |  |  |  |
| 12 | Possibility of patient experiencing adverse drug reaction present. |  |  |  |  |  |  |  |  |  |  |  |
| 13 | Prescribed product not readily available |  |  |  |  |  |  |  |  |  |  |  |
| 14 | Patient cannot afford drug product |  |  |  |  |  |  |  |  |  |  |  |
| 15 | Patient cannot tolerate the prescribed dosage form |  |  |  |  |  |  |  |  |  |  |  |
| 16 | Directions on the prescription not understood |  |  |  |  |  |  |  |  |  |  |  |
| 17 | Patient prefers not to take the prescribed medications |  |  |  |  |  |  |  |  |  |  |  |

**THANK YOU FOR YOUR TIME**
